# Deciphering why *Salmonella* Gallinarum is less invasive in vitro than *Salmonella* Enteritidis

**DOI:** 10.1186/s13567-014-0081-z

**Published:** 2014-08-30

**Authors:** Aurore Rossignol, Sylvie M Roche, Isabelle Virlogeux-Payant, Agnès Wiedemann, Olivier Grépinet, Jennifer Fredlund, Jérôme Trotereau, Olivier Marchès, Pascale Quéré, Jost Enninga, Philippe Velge

**Affiliations:** Institut National de la recherche Agronomique, UMR1282 Infectiologie et Santé Publique, F-37380 Nouzilly, France; Université François Rabelais, UMR1282 Infectiologie et Sante Publique, Tours, France; Institut Pasteur, Unité “Dynamique des Interactions Hôte Pathogène”, F-75724 Paris, France; Centre for Immunology and Infectious Disease, Blizard Institute, Barts and The London School of Medicine and Dentistry, Queen Mary University of London, London, E1 2AT UK

## Abstract

*Salmonella* Gallinarum and *Salmonella* Enteritidis are genetically closely related however associated with different pathologies. Several studies have suggested that *S*. Gallinarum is less invasive in vitro than *S*. Enteritidis. In this study we confirm that the *S*. Gallinarum strains tested were much less invasive than the *S*. Enteritidis strains tested in cells of avian or human origin. In addition, the *S.* Gallinarum T3SS-1-dependent ability to invade host cells was delayed by two to three hours compared to *S.* Enteritidis, indicating that T3SS-1-dependent entry is less efficient in *S.* Gallinarum than *S.* Enteritidis. This was neither due to a decreased transcription of T3SS-1 related genes when bacteria come into contact with cells, as transcription of *hilA, invF* and *sipA* was similar to that observed for *S.* Enteritidis, nor to a lack of functionality of the *S.* Gallinarum T3SS-1 apparatus as this apparatus was able to secrete and translocate effector proteins into host cells. In contrast, genome comparison of four *S*. Gallinarum and two *S*. Enteritidis strains revealed that all *S*. Gallinarum genomes displayed the same point mutations in each of the main T3SS-1 effector genes *sipA*, *sopE*, *sopE2*, *sopD* and *sopA.*

**Electronic supplementary material:**

The online version of this article (doi:10.1186/s13567-014-0081-z) contains supplementary material, which is available to authorized users.

## Introduction

*Salmonella* Enteritidis (SE) and *Salmonella* Gallinarum (SG) are responsible for very different infections in poultry. SE causes transient systemic infection and asymptomatic intestinal carriage, whereas SG shows poor intestinal invasion but is responsible for typhoid fever associated with a high mortality rate [1]. Furthermore, SE is able to infect plants and different species of warm and cold blooded animals, whereas SG is highly restricted to avian species. Nevertheless, these two serotypes are closely related genetically and present 99.7% homology between orthologous genes [2]. To date, despite their genetic similarities, reasons for their different pathological behavior are still poorly understood even though some differences at the genomic and proteomic levels have been described [3,4].

SG displays several distinct features suspected in part to be responsible for its distinct infection phenotype*.* The absence of type 1 fimbriae [5] and flagella could explain a reduced pro-inflammatory response compared to SE, facilitating systemic organ colonization from the gut [6,7]. Moreover, SG has lost many metabolic pathways, such as 1,2-propanediol degradation and ornithine decarboxylation, leading to restriction of usable carbon and energy sources [2]. These limited metabolic capabilities could explain SG’s reduced ability to colonize the gut, but not why it exhibits a systemic lifestyle compared to SE*.*

Two studies have suggested that SG displays a deficiency for invasion of epithelial cells compared to SE. Setta et al. demonstrated that SG strain 287/91 was about 60 times less invasive than the SE strain P125109 in chicken kidney cells [8]. Similarly Barrow and Lovell showed that the SG strain 9 was ten times less invasive than an SE strain in Vero cells [9]. However, reasons for this invasion deficiency compared to SE remain unknown.

*Salmonella* harbors several virulence factors allowing invasion of non-phagocytic cells and is the sole bacterium described as being able to enter cells by either a Zipper or a Trigger mechanism [10]. Rck, which is absent on the SG genome [11], is an outer membrane protein mediating the Zipper entry mechanism [12]. PagN is another invasin present in both SG and SE strains [13]. However, both invasins are not expressed in cell culture media [12]. The Trigger entry mechanism involves the well-characterized Type Three Secretion System 1 (T3SS-1) encoded by the *Salmonella* Pathogenicity Island-1. This secretion apparatus delivers effector proteins directly into the host cytosol after contact with the cell. Some effectors, such as SipA, SipC, SopB, SopE and SopE2, trigger extensive rearrangements of the actin cytoskeleton leading to marked membrane ruffling and bacterial internalization [14]. Some others, for example SopD or SopA, contribute to a variety of post-invasion processes, such as vacuolar development (reviewed in [15]) and modulation of the inflammatory response [16,17].

The aim of this study was to investigate why SG is less invasive in vitro than SE and whether this difference is related to the functionality of the T3SS-1. We demonstrated that SG’s low invasion ability was related to a delayed T3SS-1 entry mechanism despite the presence of a functional T3SS-1 apparatus and a similar expression of T3SS-1-related genes compared to SE.

## Materials and methods

### Bacterial strains and plasmids

*Salmonella* Enteritidis strains 02–4232, En9, ST180 and SG vaccine strain 9S were provided by the WHO Collaborating Center for *Salmonella* (Pasteur Institute, Paris, France). SE strains 1630–02, 01–7587 and SG strains 2210 and 7285 were obtained from the French Laboratory for Food safety (ANSES, Maisons-Alfort, France). SE strain LA5 was provided by the Veterinary Laboratories Agency (Addlestone, UK) [18], while SG strains 12B, 287/91 and 9 were provided by INRA Centre Val de Loire, France, the National Collection of Type Cultures (NCTC 13346) and by P. Barrow’s lab (Nottingham, UK) respectively. The genome sequence of strains LA5 (CAGR00000000), P125109 (AM933172), 287/91 (AM933173) and 9 (CM001153 to CM001154) are available on line [2,19,20]. When unspecified, bacteria were grown in Tryptic Soy Broth (TSB, Difco, Fisher-003, Illkirch, France) medium at 37 °C with shaking. Plasmids used in this study and their characteristics are listed in Additional file 1.

### Cell lines and culture conditions

Human intestinal cell line HT-29 (85061109 ECACC, Salisbury, UK), human cervical cell line HeLa (ATCC CRM-CCL-2), chicken hepatoma cell line LMH (ATCC CRL-2117), chicken lung epithelial cells CLEC213 [21] and chicken fibroblast cell line DF-1 [22] were grown in the different recommended cell culture media. Cells were routinely grown in 75 cm^2^ plastic tissue culture flasks at 37 °C under 5% CO_2_. Throughout this study, cells were grown without antimicrobial compounds.

### Adhesion/invasion assays

Adhesion-invasion assays were performed as previously described [23]. Briefly, 24-well tissue culture plates were seeded five days before infection in order to obtain confluent monolayers for the day of infection. Bacteria were grown overnight in TSB medium at 37 °C without shaking. Each experiment used two plates: one to determine the total number of adhered and invaded bacteria (adhesion/invasion assay) and the second the invaded bacteria only (invasion assay). Cell monolayers were incubated in medium without antibiotics for 24 h and then infected for different times (0.5 h, 1 h, 1.5 h, 3 h or 4.5 h) at 37 °C with 10^7^ CFU in 300 μL (multiplicity of infection 10). For adhesion/invasion assays, the cell monolayers were gently washed six times with phosphate buffered saline (pH 7.3) and then disrupted with 1 mL cold distilled water (4 °C). Viable bacteria (intra- and extracellular) were counted after plating serial dilutions on TSA (Difco). For the invasion assay, the second plate was washed with Dulbecco’s modified Eagle’s medium (DMEM, Life Technologies, Fisher-003, Illkirch, France) and incubated in culture medium containing 100 μg of gentamicin per mL. After 1.5 h at 37 °C, cells were washed with phosphate-buffered saline and lysed with 1 mL cold distilled water (4 °C). Viable intracellular bacteria were assessed by serial dilutions plated on TSA. Cell viability was checked in parallel to infection by the trypan blue exclusion test. The results are expressed as the percentage of CFU recovered relative to the number of bacteria deposited per well. Experiments were carried out in duplicate and repeated at least twice for each strain.

### Construction of LA5*invA* and 287/91*invA* mutants

The Datsenko and Wanner method [24] was used to construct SE LA5*invA* and SG 287/91*invA* mutants with two modifications. Firstly, 35 mL of LB medium (Difco) containing 100 μg.mL^−1^ carbenicillin were seeded with 350 μL of an overnight culture of strains SE LA5 or SG 287/91 carrying pKD46 and incubated at 30 °C without shaking. One hour before OD_600nm_ reached 0.3, the medium was supplemented with 5 mM arabinose (final concentration). Secondly, bacteria were washed three times with 10% glycerol water supplemented with 1 mM 3-morpholinopropane-1-sulfonic acid buffer and were finally resuspended in 100 μL of GYT medium (10% glycerol, 0.125% yeast extract (Difco) and 0.25% tryptone (Difco)). Kanamycin (50 μg/mL) or chloramphenicol (30 μg/mL) resistant mutants for SE LA5 and SG 287/91 respectively were selected and checked using PCR.

### Secretion assays

In order to analyze the T3SS-1 secretion profile of our strains, in vitro secretion assays were performed as previously described, after culture of bacteria in 30 mL of LB broth containing 0.3 M NaCl, a medium inducing SPI-1 gene expression [25]. Bacteria were grown until the OD reached 1.8-2.0 and then cultures were harvested by centrifugation for 15 min at 7000 *g* at 4 °C. Culture supernatants were filtered through 0.2 μm filters. Secreted proteins were precipitated by adding trichloroacetic acid (10% final) and an incubation at 4 °C for 30 min. Proteins were recovered by centrifugation at 12 000 *g* for 20 min and resuspended in 100 μL of Laemmli buffer. Samples were boiled for 5 min at 99 °C. β-lactoglobuline was added to each culture supernatant to control for protein precipitation and sample loading. Proteins were separated using SDS-PAGE (10% acrylamide), and then stained with Coomassie brilliant blue G-250 [26]. Gels were visualized with a Fusion FX7 imaging system (Vilber Lourmat).

### Translocation assay

A β-lactamase (TEM-1) translocation assay of effector proteins was performed as previously described by Fookes et al. [27]. SG 287/91 wild-type and its *invA* mutant strain carrying TEM-1 fusions were incubated for 3 h with HeLa and CLEC213 cells at MOI 100. Translocation was detected by fluorescence microscopy (Leica) using fluorescent CCF4/AM β-lactamase substrate (LiveBLAzer FRET-B/g Loading Kit, Invitrogen, Fisher-003, Illkirch, France).

### Actin foci quantification

HeLa cells (1 × 10^5^) were seeded two days before infection in 24-well plates on 12 mm microscope cover glasses. The day before infection, 1 μg of pEGFP-actin was used to transfect cells using Nanofectin as recommended by the manufacturer (PAA-The Cell Culture Company, PAA Labora, Les Mureaux, France). After 3 h, fresh serum containing medium was added for 16 h and the cells were then antibiotic-starved. After three washes, cells were incubated at MOI 50 with mCherry labeled bacteria, i.e. carrying the pFPVmcherry plasmid [28] (Additional file 1), which were grown overnight in LB broth medium at 37 °C without shaking. After centrifugation for 5 min at 50 *g* at room temperature and 0.5 h at 37 °C, the cells were washed three times and fixed in PBS 4% paraformaldehyde at room temperature. Coverslips were mounted in fluorescence mounting medium and analyzed using an Apotome system (Zeiss). Actin foci associated with each strain were scored visually for 100 cells labelled with actin-EGFP.

### Quantification of intracellular bacteria replication

**Figure 1:**
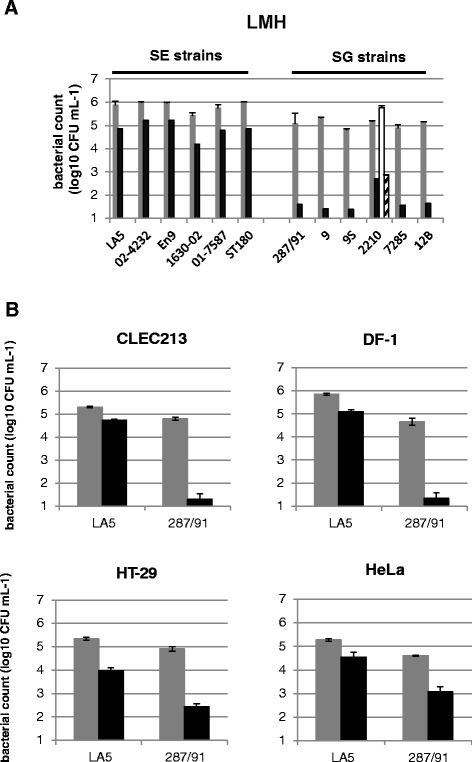
***S.*** **Gallinarum is less invasive in vitro than** ***S.*** **Enteritidis regardless of the cell origin. A**. Invasive ability of six *S.* Enteritidis and six *S.* Gallinarum strains were compared using gentamicin protection assays performed with LMH cells. Bacteria (MOI 10) were deposited on cells for 0.5 h. Grey bars represent the number of adhered and intracellular bacteria recovered after washings and black bars the number of intracellular bacteria only. White bar represents the number of adhered and intracellular bacteria and hatched bars the number of intracellular bacteria obtained for SG 2210 strain when the cell plate was centrifuged immediately after infection. The results correspond to the mean ± standard deviation of three independent experiments performed in duplicate and are expressed in log_10_ CFU mL^−1^. Similar results were reproduced with other cell lines. **B**. Invasive ability of *S.* Enteritidis LA5 and *S.* Gallinarum 287/91 strains were compared using gentamicin protection assays performed in avian (CLEC213 and DF1 cells) and human cell lines (HT-29 and HeLa cells). Bacteria (MOI 10) were deposited on cells for 0.5 h. Grey bars represent the number of adhered and intracellular bacteria and black bars the number of intracellular bacteria. Results correspond to the mean ± standard deviation of two independent experiments performed in duplicate and are expressed in log_10_ CFU mL^−1^. Similar results were obtained with other *S.* Gallinarum and *S.* Enteritidis strains.

CLEC213 cells were seeded in 24-well plates on 12 mm microscope cover glasses to obtain 90% confluent monolayers on the day of infection. Cells were infected for 0.5 h or 3 h with mCherry-labeled strain LA5 at MOI 100 or strain 287/91 at MOI 1000 to obtain the same amount of adhered bacteria per dish for the two strains according to the results presented in Figure 1. After 1.5 h at 37 °C in medium containing 100 μg.mL^−1^ gentamicin infection, the cells were washed three times and fixed in PBS 4% paraformaldehyde. Extracellular bacteria were successively stained for 1 h with rabbit anti-*Salmonella* serum (monovalent O:9, dilution 1:100, BioRad, Marnes La Coquette, France) and Alexa488-coupled goat anti-rabbit antibodies (dilution 1:1000, Molecular Probes, Leiden, NLD) after 45 min at room temperature in PBS 1% BSA. As described, after entry, *Salmonella* reside inside a *Salmonella* containing vacuole (SCV), thus grouped intracellular bacteria were assumed to be located within the same vacuole. The number of bacteria per vacuole was therefore counted for 100 vacuoles using an Apotome system (Zeiss).

### Quantification of bacterial replication in the cellular culture medium

Bacteria were grown overnight in TSB at 37 °C without shaking. Prior to the infection, bacteria were diluted in DMEM-F12 medium (Invitrogen). Bacteria (1.2 × 10^7^ per well) were distributed in 24-well non-seeded plates and were incubated for 1.5 h, 3 h and 4.5 h at 37 °C under 5% CO_2_. For each time point, bacterial multiplication in the cellular culture medium was determined by plating serial dilutions on TSA.

### Measurement of *invF*, *sipA* and *hilA* mRNA levels

Expression of *invF*, *sipA* and *hilA* was measured using semi-quantitative Reverse Transcription PCR. CLEC213 cells were seeded in 100 mm diameter dishes to obtain confluent monolayers on the day of infection. Cells were infected for 0.5 h with strain SE LA5 at MOI 100 or strain 287/91 at MOI 1000 in order to obtain the same amount of adhered bacteria per dish for the two strains. CLEC213 were lysed using a 0.1% SDS, 1% acidic phenol, 19% ethanol mixture [29] and a bacterial pellet was obtained through centrifugation. Bacterial RNA was purified using the RNeasy Mini Kit (Qiagen, Courtaboeuf, France) and genomic DNA contamination was removed using the TURBO DNA-free kit, in accordance with the manufacturer’s instructions (Ambion, Spitfire Close-Ermine Business, UK). Bacterial RNA was reverse transcribed to cDNA in the presence of random hexamers using the SuperScript III First-Strand Synthesis SuperMix (Invitrogen) and PCR amplifications were performed using the appropriate primers (Additional file 1). Reactions without reverse transcription were performed to check the lack of DNA contamination in the RNA preparation. Expression of the housekeeping gene *tufA* was also measured as a control for bacterial RNA quantity. PCR samples were loaded on an agarose gel and band intensity was assessed using Quantity One software (BioRad).

### Statistical analysis

Unpaired t test with Welch’s correction (because variances are significantly different) was used with the number of CFU as a variable to analyze the differences in the invasion assays. The Gaussian distribution of our values was tested by the d’Agostino and Pearson omnibus normality test. Data were analyzed using Graph Pad Prism software 5.0.

## Results

### *S*. Gallinarum is less invasive in avian epithelial cells in vitro than *S*. Enteritidis

In order to confirm the low invasive phenotype of SG compared to SE, the invasive ability of six strains of each serotype were compared using adhesion/invasion assays performed in avian hepatoma cells (LMH) (Figure 1A). On average SG strains were 1500-fold less invasive than SE strains after 0.5 h of bacteria-host cell contact (1.97 ± 0.41 versus 5.17 ± 0.38 log_10_ CFU respectively). An unpaired t test showed that this difference was highly significant (*p* < 0.0001). These data suggest that for all the strains tested SG was less invasive than SE. Figure 1A shows that on average the SG strains were six times less adhesive than the SE strains (5.07 ± 0.19 versus 5.88 ± 0.22 log_10_ CFU respectively (*p* < 0.0001)), suggesting that the marked invasion deficiency of SG could not be explained by a deficiency in adhesion alone. The lack of a link between poor adhesion and poor invasion observed for SG was confirmed by centrifuging the plate immediately after infection using the SG strain 2210 as an example (Figure 1A, white bar). Centrifugation enabled the main part of the bacterial population to come rapidly into contact with cells independently of an adhesion defect or lack of motility. Under this condition, the number of SG that adhered was four times higher (white bar) than for non-centrifuged bacteria, however, the number of intracellular bacteria (hatched bar) remained unchanged, confirming that the adhesion defect did not explain the invasion defect.

### *S*. Gallinarum is less invasive in vitro than *S*. Enteritidis regardless of the cell origin

We previously observed that the SG strains tested were less invasive in avian cells than the SE strains. To determine whether their low invasive phenotype depends on the cell origin, adhesion/invasion assays were performed using other avian (CLEC213 and DF-1) and human (HeLa and HT-29) cell lines (Figure 1B). For practical reasons, further experiments were performed using only one representative strain of each serotype: the SE LA5 strain, responsible for transient systemic infection and intestinal carriage in the chicken and the SG 287/91 strain, responsible for lethal systemic infection in poultry. The results show that the SG 287/91 strain was always significantly less invasive than the SE LA5 strain (30- to 4000-fold, depending on the cell line tested), demonstrating that SG is less invasive in vitro than SE regardless of the cell origin. As previously observed, Figure 1B shows that SG displayed an adhesion defect compared to SE in all the cell lines tested, but the difference was insufficient to explain the invasion defect. Moreover, no correlation was observed between adhesion and invasion rates.

It is interesting to note that the SG 287/91 strain was significantly less invasive for avian than for human cell lines (*p* < 0.0001) (less than 10 intracellular bacteria compared to 70 for 1 × 10^7^ bacteria deposited respectively). This was not observed with the SE LA5 strain, which displayed similar invasive abilities regardless of the origin of the cell line. Further experiments are required to explain this phenotype.

### *S*. Gallinarum exhibits a functional T3SS-1 apparatus but is unable to induce membrane ruffles

As the T3SS-1 is the main invasion factor known in *Salmonella*, we investigated further whether a dysfunctional T3SS-1 apparatus could explain the low invasive ability of SG. The analysis of the genomes of the four SG strains 287/91, 2210, 9 and 9S revealed that all T3SS-1 genes as well as all effectors encoding genes known to be secreted by the T3SS-1 are present in SG genomes (data not shown).

**Figure 2:**
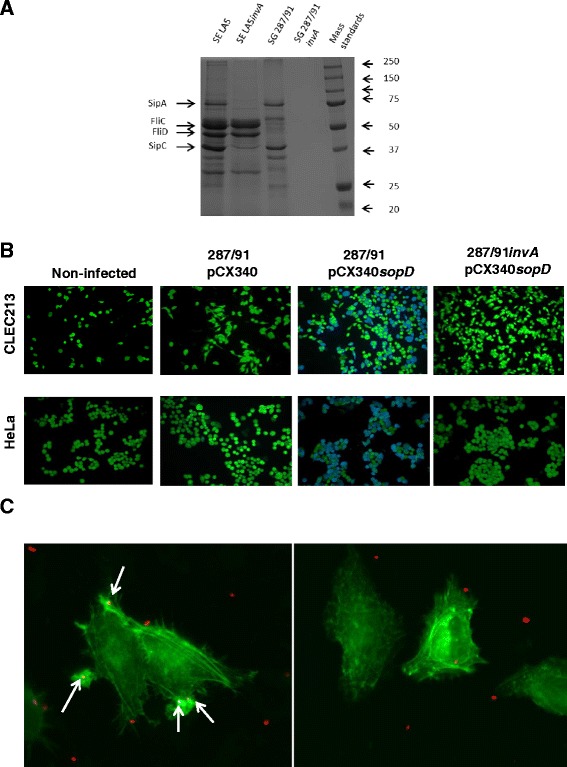
***S*** **. Gallinarum’s invasion defect is related to a T3SS-1 defect in spite of a functional T3SS-1 apparatus. A**. Strain SG 287/91 secretes proteins in the culture medium through its T3SS-1. Secretion assays were performed in LB NaCl 0.3 M. Proteins secreted in the supernatant by SE LA5, SG 287/91 and their T3SS-1 isogenic mutants were precipitated, separated using SDS-PAGE and stained with Neuhoff blue. The experiment was performed three times. **B**. Strain SG 287/91 translocates SopD effector into the host cell cytosol through its T3SS-1. Translocation assays were performed using strains SG 287/91 and 287/91*invA* carrying a plasmid encoding a fusion SopD-TEM-1 β-lactamase (pCX340sopD). When SopD was translocated into the cells (avian CLEC213 or human HeLa cells), β-lactamase hydrolyzed a green fluorescent substrate previously loaded in the cells (CCF4/AM), releasing a blue fluorescent substrate. The experiment was performed three times. **C**. Strain SG 287/91 is associated with less actin foci than strain SE LA5. Fluorescent SE LA5 or SG 287/91 bacteria were deposited on HeLa EGFP-actin-transfected cells for 0.5 h with an MOI of 100 and 1000 respectively to obtain a similar number of adhered bacteria. White arrows indicate actin foci associated with bacteria. The number of actin foci associated with bacteria per 100 transfected cells was determined visually using an Apotome system microscope. The experiment was performed in triplicate. Photographs illustrate the impact of *S.* Enteritidis (left) and *S.* Gallinarum (right) on the actin cytoskeleton.

To determine whether the T3SS-1 was functional, the secretion profiles of these serotypes were compared in a medium inducing T3SS-1 expression as previously described [30]. Proteins corresponding to FliC and FliD flagellar proteins were absent in the SG 287/91 strain secretion profile compared to that of the SE LA5 strain, which is consistent with the aflagellated status of SG. In contrast, both secretion profiles contained proteins whose sizes corresponded to T3SS-1 effectors SipA and SipC (Figure 2A). These proteins were absent from the secretion profile of *invA* mutants, confirming that they were secreted in a T3SS-1-dependent manner in wild-type strains. These data demonstrate that SG is able to secrete at least some effector proteins in the culture medium through its T3SS-1 apparatus.

To determine whether SG was able to translocate T3SS-1 effectors into the host cell cytosol, a fluorescence-based translocation assay was performed using a fusion protein of the effector SopD with mature TEM-1 β-lactamase and fluorescent β-lactamase substrate CCF4. The emission of blue fluorescence by eukaryotic cells previously loaded with the substrate reveals the cleavage of the CCF4 substrate by β-lactamase and therefore the translocation of SopD into these cells. Uncleaved CCF4 emits green fluorescence. Figure 2B shows that blue cells were observed when avian (CLEC213) and human (HeLa) cells were infected with the SG 287/91 strain carrying pCX340*sopD*, the plasmid encoding SopD-TEM-1, demonstrating that SG was able to translocate the fusion protein into the cytosol of both avian and human cells. In contrast, cells infected with SG 287/91 carrying the empty vector pCX340 emitted a green fluorescence, indicating that conversion of the fluorescence of CCF4 was only due to the presence of the fusion protein in the host cell. This translocation was T3SS-1-dependent since the 287/91*invA* strain carrying pCX340*sopD* was not able to translocate SopD-TEM-1 into the host cell cytosol. These data demonstrate that the SG 287/91 strain was able to translocate at least the SopD effector through its T3SS-1 apparatus into the cytosol of host cells and thus that the T3SS-1 apparatus of SG was functional.

Finally, since the SG 287/91 strain harbors a fully functional T3SS-1 apparatus, and since cell invasion by T3SS-1 leads to strong actin reorganization, we investigated the impact of SG T3SS-1-dependent entry on the host cell actin cytoskeleton. The quantification of actin foci only takes into account the Trigger mechanism, which mediates extensive actin rearrangements in contrast to the Zipper mechanism [10]. Actin-GFP transfected cells were used in order to visualize the actin reorganization occurring during cell invasion mediated by the T3SS-1 using fluorescence microscopy (Figure 2C). Unlike the other cell lines tested, HeLa cells displayed a good transfection rate and were therefore used to perform these experiments. Quantification of actin foci shows that after 0.5 h of infection the SG 287/91 strain was associated with 50-fold less actin foci than the SE LA5 strain (0.7 ± 0.6 for 100 cells vs 34.3 ± 13.3, respectively), strongly suggesting that the SG invasion defect is mainly related to a T3SS-1 invasion defect.

### *S*. Gallinarum’s T3SS-1 invasive ability increases with time

**Figure 3:**
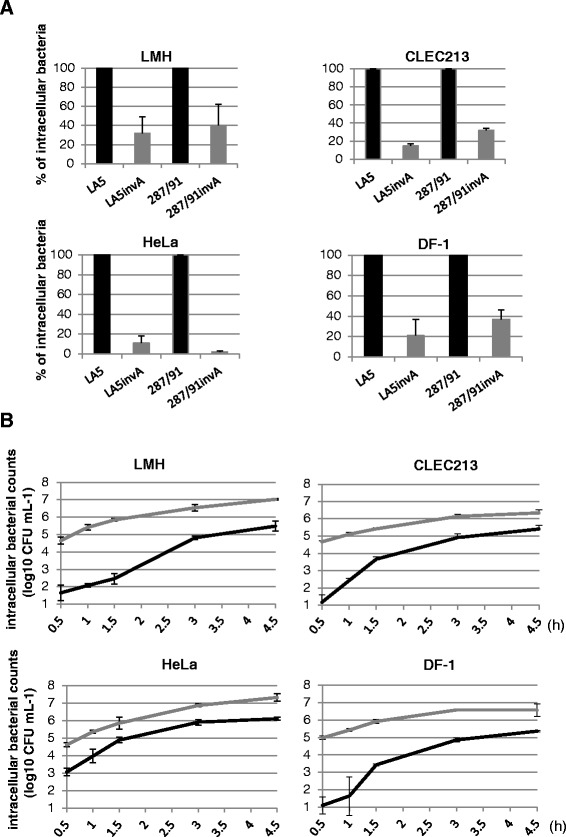
***S.*** **Gallinarum and** ***S.*** **Enteritidis mainly invade cells through a T3SS-1-dependent pathway but** ***S.*** **Gallinarum invasive ability is delayed compared to** ***S.*** **Enteritidis. A**. T3SS-1 inactivated mutants of *S*. Gallinarum and *S*. Enteritidis show the same relative entry defect. Gentamicin protection assays were performed in avian (LMH, CLEC213, DF-1) and human (HeLa) cell lines. Infection was carried out for 0.5 h with wild-type strains (black bars) and their *invA* mutants (grey bars). Results obtained for the *invA* mutants are expressed relative to values obtained for the wild-type strains, arbitrarily set at 100%. Raw data are presented in Additional file 2. Values represent means ± standard deviation of two independent experiments performed in duplicate. **B**. *S*. Gallinarum’s T3SS-1 dependent invasive ability is delayed in avian cell lines compared to *S*. Enteritidis. Gentamicin protection assays were performed in avian (LMH, CLEC213, DF-1) and human (HeLa) cell lines. Infection was carried out for 0.5 h to 4.5 h using SE LA5, SG 287/91 strains and their respective *invA* mutants. To assess the role of the T3SS-1, the number of intracellular bacteria recovered for the mutant strain was subtracted from the number recovered from the wild-type strain at each time point. The grey line represents T3SS-1-dependent entry of the SE LA5 strain and the black line represents that of the strain SG 287/91. The results correspond to the mean ± standard deviation of two independent experiments performed in duplicate and are expressed in log_10_ CFU mL^−1^.

SG was associated with less actin foci than SE, suggesting that SG is defective for T3SS-1 invasion, despite having previously demonstrated that its T3SS-1 was functional. To investigate this further, the impact of an *invA* mutation on SG’s invasive ability was analyzed. This mutation hampers the formation of the T3SS-1 apparatus (Figure 3 and raw data Additional file 2). To compare the respective roles of the T3SS-1 for SG and SE invasion, the results obtained for the *invA* mutants are expressed in relation to values obtained for the wild-type strains, arbitrarily set at 100%. Thus, even though the T3SS-1 is defective in SG, the entry level of the *invA* mutant will be similar to the wild-type strain. Figure 3A shows that, after 0.5 h of contact between bacteria and host cells, the entry level of the SG 287/91 *invA* mutant was reduced compared to the wild-type strains (between 2.5 and 50 times lower, depending on the cell line used). This result demonstrates that the majority of the wild type SG 287/91 bacteria invaded cells using their T3SS-1. Unexpectedly, similar percentages were obtained with the SE LA5 strain suggesting that SG uses its T3SS-1 to invade non-phagocytic cells in the same proportion as SE does (Figure 3A). It is thus surprising that beside the fact that SG can use its T3SS-1, the raw number of intracellular SG observed was much lower than for SE after 0.5 h of bacteria-host cell contact (Figure 1 and Additional file 2). One hypothesis could be that the SG’s T3SS-1 was less efficient than that of SE.

In order to investigate the time course of the invasion process, invasion kinetics was studied using the wild-type strains and their *invA* mutants (used already in Figure 3A). To determine the contribution of the T3SS-1 over time, the number of intracellular bacteria obtained for each *invA* mutant was subtracted from the number obtained for the corresponding wild-type strain (Figure 3B, raw data Additional file 2). Experiments were carried out in avian (LMH, CLEC213 and DF-1) and human cell lines (HeLa and HT-29). The results show that after 0.5 h, the SG 287/91 T3SS-1-dependent invasion ability was dramatically impaired compared to that of the SE LA5 strain (30- to 5000-fold lower according to the cell line tested). For between 1 h and 4.5 h of interaction, intracellular number of bacteria increased over time for both strains in all tested cell lines , due to longer bacteria-host cell contact time, but the results for SG remained lower. However, the number of intracellular bacteria increased much more strongly with SG than with SE and particularly in avian cells. Indeed, in avian cells, SG’s T3SS-1 invasion abilities increased by 15 to 150 times more than that observed for SE from 0.5 to 3 h. This increase in the number of intracellular SG over time was also observed for human cells but to a lower extent.

A possible explanation of this result is that SG can use its T3SS-1 apparatus, but its T3SS-1 dependent invasive ability is delayed compared to that of SE, in particular in avian cells. However, as we performed assays with long infection periods, the increase in the number of intracellular bacteria could also be related to intracellular replication of bacteria. To investigate this hypothesis we measured the number of intracellular bacteria per vacuole over time using fluorescence microscopy, as described in the Materials and methods. Table 1 shows that for the SE LA5 strain, only one or two bacteria were observed per vacuole for 98% of the intracellular bacteria until 3 h of bacteria-host cell contact. After that, the number of bacteria per vacuole increased, suggesting that SE started to replicate intracellularly. For the SG 287/91 strain, the number of bacteria per vacuole was higher as early as 1 h after bacteria-host cell contact since 8% of the vacuoles contained more than five bacteria at that time. After 1 h of infection, a higher intracellular number of SG than of SE can be attributed to the high MOI (1000) used to observe sufficient intracellular SG in these experiments. This was demonstrated by the fact that the same phenomenon was observed for the SE LA5 strain at an MOI of 1000 (data not shown). Nevertheless, the number of SG per vacuole after 3 h and 4.5 h of infection was not very different from that at 1 h (14 and 9% respectively), suggesting that SG replicated slowly within cells during this period. These results demonstrate that the increase in the number of intracellular bacteria observed between 0.5 h and 3 h was not related to intracellular replication.

**Table 1:** **Increase in the number of intracellular bacteria between 0.5 h and 3 h is not related to replication of intracellular bacteria**

**Strain**	Time of bacteria-cells contact*	**Number of bacteria per vacuole**			
		**1-2**	**3-5**	**6-10**	**≥ 10**
**LA5**	**1 h**	**98** ^**§**^	**1**	**1**	**0**
**287/91**		**84**	**8**	**6**	**2**
**LA5**	**3 h**	**99**	**1**	**0**	**0**
**287/91**		**78**	**8**	**10**	**4**
**LA5**	**4.5 h**	**84**	**9**	**4**	**3**
**287/91**		**81**	**10**	**5**	**4**

*Gentamicin protection assays were performed in CLEC213 cells using mCherry labeled bacteria, at MOI 100 for LA5 and MOI 1000 for 287/91 to obtain a sufficient number of intracellular bacteria. Extracellular bacteria were killed with 100 μg.mL^−1^ gentamicin and stained in green in order to differentiate extracellular and intracellular bacteria through fluorescent microscopy.

^§^Number of bacteria per vacuole was counted for 100 vacuoles. The experiment was performed three times. Results correspond to the mean percentage of vacuoles containing 1–2, 3–5, 6–10 or ≥ 10 bacteria.

Bacterial replication in the cell culture medium during the infection phase could also explain the increase in the number of intracellular bacteria between 0.5 h and 3 h by artificially increasing the MOI. To test this hypothesis, bacterial replication rates were assessed in the cell culture medium over time. The results show that SG replicated more slowly than SE in the cell culture medium, as previously described in bacterial culture medium. Indeed, the SG 287/91 bacteria number increased only 25-fold between t_0_ and 3 h, while the SE LA5 bacteria number increased 150 times in cell culture medium. This result shows that the increase in intracellular SG 287/91 was not related to this serotype having a higher replication rate than SE in the cell culture medium during the infection period.

Overall, these data demonstrate that the greater increase in the number of intracellular SG than of SE was not related to a higher replication rate but to an increase in T3SS-1-dependent invasive ability over time.

### *S*. Gallinarum SPI-1 gene expression is similar to *S*. Enteritidis SPI-1 gene expression and *S*. Gallinarum effectors contain point mutations

**Figure 4:**
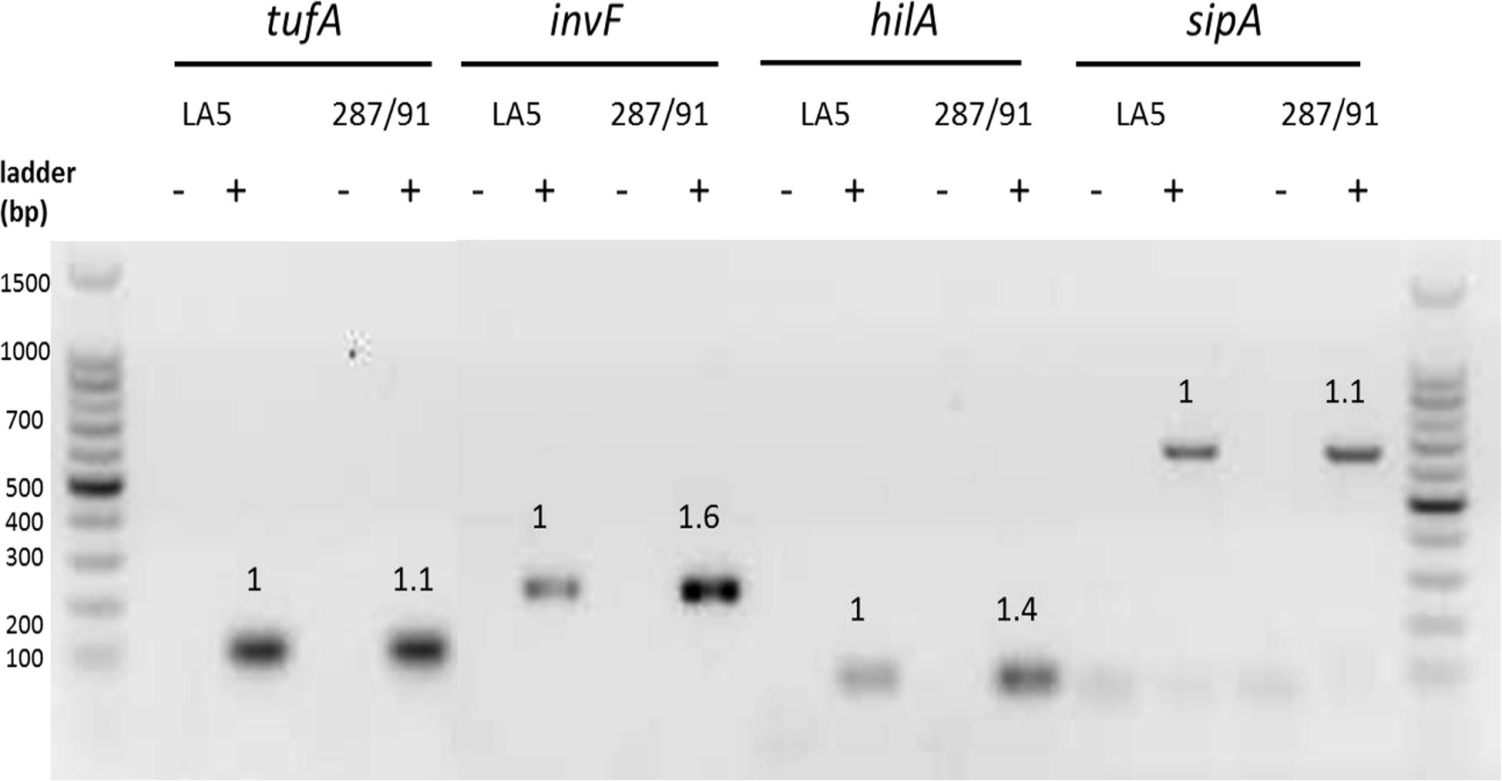
***S*** **. Gallinarum’s invasion defect is not related to a T3SS-1 gene-expression defect.** RNA from LA5 and 287/91 strains were collected after 0.5 h of contact with CLEC213 and were reverse transcribed. *sipA*, *hilA* and *invF* cDNA was amplified using PCR after DNase treatment and reverse transcription (+) or not (−). Housekeeping gene *tufA* expression was measured as a control for RNA quantity. Band intensity was quantified using Quantity One software (BioRad). The number above each band represents the ratio of SG287/91 divided by SE LA5 intensities for each gene. Three independent experiments were performed.

The delay in the T3SS-1-dependent invasion observed for SG could be related to a lower T3SS-1 gene expression. To test this hypothesis, we measured the expression of *hilA* (the main transcriptional regulator of T3SS-1 apparatus expression), *invF* (a positive regulator of T3SS-1 effector expression) and *sipA* (an effector protein) after 0.5 h of contact with avian cells (CLEC213) through reverse transcription and semi-quantitative PCR. The results show that the SG 287/91 strain expressed these genes at a similar level to the SE LA5 strain (Figure 4), demonstrating that the invasion defect observed for SG after 0.5 h of bacteria-host cell contact was not related to a lower expression of T3SS-1 related genes.

As SG was shown to display a functional T3SS-1 apparatus and correctly expresses both the T3SS-1 apparatus and T3SS-1 effector genes, our data suggests that the delay in SG invasion could be due to dysfunctional T3SS-1 effector proteins. An in silico analysis of the genomes of 6 *Salmonella* strains (2 SE and 4 SG) identified identical point mutations in the main T3SS-1 effectors (SipA, SopA, SopD, SopE and SopE2) in all the SG strains but not in SE (Table 2). These point mutations led to an early stop codon being introduced in *sopA* and to amino acid substitutions in the other effectors. Moreover, these considerable modifications of amino acids could affect the protein stability or the protein functionality (Table 2). For example two mutations in SipA (Ala125/Glu and Gln260/Lys) could modify the chaperone binding domain activity. Other mutations were also observed in all the SG strains analyzed: i) in the guanine exchange nucleotide factor domain of SopE and SopE2 and ii) in SopD (Ile60/Thr and Asn307/Lys).

**Table 2:** ***S*** **. Gallinarum T3SS-1 effectors display potential causal mutations**

**Effector**	**Position of mutation**	**Domain**	***S.*** **Enteritidis**		***S.*** **Gallinarum**			
			**P125109**	**LA5**	**287/91**	**2210**	**9**	**9S**
SipA	13	-	Met		Ile			
	125	Chaperone-binding site	Ala		Glu			
	260	Chaperone-binding site	Gln		Lys			
	372	-	Thr		Met			
SopA	446	-	length: 783 aa		length: 446 aa			
SopD	60	-	Ile		Thr			
	307		Asn		Lys			
SopE	237	GEF catalytic domain	Gln		Pro			
SopE2	152	GEF catalytic domain	Glu		Gly			

In order to determine whether one of these effectors was inactive in SG, an attempt to complement the SG 287/91 strain with one of the corresponding genes of the invasive strain SE LA5 was carried out. However, single complementation did not increase the invasive phenotype of the SG strains, suggesting that either the mutations have no deleterious impact on the invasive phenotype or, more probably, that more than one mutated effector is responsible for the low invasive ability of SG (Additional file 3). Further studies should be performed to make the point with different polarized and non-polarized cell lines because it has been described that the T3SS-1 dependent entry process is dependent on used cell lines and polarization [31,32].

## Discussion

*Salmonella* is a facultative intracellular bacterium and several reports have shown that cell invasion is critical for disease to develop. Previous work performed with one epithelial cell line and one strain for each, SG and SE reported that the SG strain was less invasive than the SE strain after 0.5 h of bacteria-host cell contact [8,9]. Our work has extended this observation to six strains of each serotype and to five cell lines, strengthening the notion that SG is less invasive than SE and demonstrating that its low invasive ability is at the serotype scale. These findings were observed with cells originating from avian and human sources, suggesting that host specificity and lethal systemic spread of SG in the chicken are not related to specific invasiveness for avian non-phagocytic cells. Large metabolic networks which are intact in genomes of gastrointestinal *Salmonella* like SE but degraded in genomes of extra-intestinal serotypes and which enable growth of *Salmonella* in the inflamed gastrointestinal tract, could be involved in host specificity and disease pattern [33]. Furthermore, our in vitro results showing that SG poorly invades non-phagocytic cells suggest that enterocytes are probably not the main entry route for SG in vivo from the gut to the systemic apparatus, although it is not possible to exclude the hypothesis that a low invasion rate of these cells could be sufficient to initiate systemic infection.

In line with previous studies, we also observed that all tested SG strains were less adhesive than the SE strains [5,34]. However, this phenotype was not sufficient on its own to explain the difference in invasion between these serotypes. Indeed, SG’s adhesion rate was, on average, only six-fold lower than SE, whereas its ability to enter was up to 4000-fold lower depending on the cell line tested. Furthermore, no correlation was observed between the differences in adhesion rates and in entry levels for the five cell lines and the twelve *Salmonella* strains tested. The low invasive phenotype of SG was also observed when bacteria were in close contact with cells (after centrifugation of plates), which strongly suggests that poor invasion rates were related to a deficient entry ability.

*Salmonella* is able to enter cells through at least three invasion routes mediated by the T3SS-1, which induces extensive actin cytoskeletal rearrangements, and two invasins (PagN and Rck) which induce limited actin cytoskeletal rearrangement [10]. Rck is absent in SG [11], hence it could be thought to be responsible for its poor invasion behavior. However, Rck and PagN are not expressed in *Salmonella* under our culture conditions [12,13], suggesting that the invasion deficiency observed for SG cannot be explained by differences in these two invasins. In contrast, fluorescent microscopy of actin transfected cells shows that SG induced a very low number of actin foci compared to SE suggesting that the invasion defect could be related to a defect in the Trigger mechanism.

In *Salmonella,* the Trigger entry mechanism is mediated by the T3SS-1. Our study demonstrates that the low invasive phenotype cannot be related to an inability of SG to use its T3SS-1. Indeed, SG was able to secrete T3SS-1 effectors in vitro and to translocate them within host cells in a T3SS-1 dependent manner. However, a SG *invA* mutant unable to use its T3SS-1 was defective in entering cells compared to the wild-type strain, showing that the T3SS-1 is, at least in part, functional in SG. Finally, the kinetics of cell invasion demonstrate that SG was able to invade cells efficiently using its T3SS-1, but only after a very long contact time with host cells, whereas the invasion of SE was more efficient.

As SG harbors a functional T3SS-1 apparatus, the T3SS-1 defect could be explained by a lower SPI-1 gene expression compared to that of SE or by dysfunctional T3SS-1 effectors. The present study shows that T3SS-1 regulatory genes *hilA* and *invF* and the effector gene *sipA* were expressed at similar levels to those in SE after bacteria-host cell contact, suggesting that all T3SS-1 effectors are well expressed by SG. Moreover, whole genome comparison revealed that the four SG genomes analyzed (287/91, 9, 9S, and 2210) displayed all the known genes encoding T3SS-1 effectors. However, these genomes displayed the same point mutations in the main T3SS-1 effector genes *sopE*, *sopE2*, *sipA, sopD* and *sopA* unlike SE strains P125109 and LA5. For *sopE*, *sopE2* and *sipA*, these mutations change the amino-acid sequence. Furthermore, *sopA* has an early STOP codon in the mutated version leading to a shorter SopA protein. This mutation has been previously described by Thomson et al. [2]. All these changes could affect the proper folding of the proteins or could modify their function as they are in functional domains (Table 2). SopE and SopE2 are paralogues and act as eukaryotic guanine exchange nucleotide factors (GEF). They trigger the exchange of GDP for GTP leading to activation of Rho GTPases Rac1 and Cdc42, responsible for actin polymerization and formation of membrane ruffles [35,36]. A mutation in the GEF catalytic domain of these proteins (Table 2) could thus modify the invasive ability of SG. SipA binds actin directly and induces its polymerization by decreasing the critical concentration for actin assembly [37,38]. Two of the four mutations in SipA were present in the chaperone binding domain suggesting that SipA of SG could be weakly or slowly translocated within the host cells. Thus the mutations in the genes encoding these three proteins could explain why SG induced fewer actin foci than SE and also the delayed entry during early stages of infection, as a *S*. Typhimurium *sipA* mutant was shown to enter cells more slowly than its wild-type parent especially in polarized cells [39]. Finally, SopD plays a role in membrane fission after internalization of the bacteria [16,40] and SopA exhibits an E3 ubiquitin ligase activity in addition to its potential to modulate the intestinal inflammation response [41]. SopD and SopA are also both involved in invasion of polarized epithelial cells [16]. Overall, the present study shows that SG harbors mutations in genes encoding the main effectors which could contribute to its defective invasion.

In order to test the impact of these mutations on cell invasion, an attempt was made to complement the SG 287/91 strain with either *sipA*, *sopE* or *sopA* from the SE LA5 strain. SopE2 was not introduced due to its redundancy with SopE [42]. However, the invasive ability of our single complemented strains was not restored after 1 h of bacteria/LMH cell contact (Additional file 3). Complementation was also not observed after infection of polarized Caco-2 cells with *sipA* complemented SG strain (data not shown). This result suggests that SG’s T3SS-1 defective invasion could be related to a combination of mutations in several T3SS-1 effector genes. Another hypothesis, which is compatible with the first one, is that the lower adhesion observed with SG compared to SE hampered an intimate contact between SG and its host cells, which is necessary for the injection of T3SS-1 effectors. This hypothesis is supported by the demonstration that establishment of close contact is a prerequisite for subsequent T3SS-1-dependent invasion of some cell lines [43]. Nevertheless, we have demonstrated by β-lactamase (TEM-1) translocation assay that SG, like SE is able to inject T3SS-1 effectors. The fact that this test is not quantitative does not allow us to exclude this second hypothesis. Further experiments are required to investigate the impact of adhesion on the intimate contact of SG and the role of these mutations on the biological function of these effectors and on the pathogenicity of SG.

A current hypothesis is that ubiquitous serotypes, such as SE, elicit a strong pro-inflammatory response in the gut limiting the development of infection [44,45]. On the contrary to ubiquitous serotypes, host specific serotypes such as SG are not thought to cause this strong pro-inflammatory response, thus allowing systemic dissemination and severe disease [6,46]. Several bacterial antigens including T3SS-1 effectors have been shown to elicit the pro-inflammatory response. Wood et al. have shown that *S.* Dublin SopA induces fluid secretion and polymorphonuclear cell influx in a bovine ligated loop model [47]. An SE *sipA* mutant has also been shown to impair its capacity to promote the expression of two pro-inflammatory chemokines CXCLi1 and CXCLi2, [48]. Further work is necessary to determine whether the T3SS-1 effector mutations identified in our study can help SG to escape the pro-inflammatory response.

It is interesting to note that, like SG, both *S.* Typhi and *S.* Abortusovis have a low entry rate into host cells and are host specific and induce systemic infection. To determine the relationship between the respective invasion rates and induction of systemic infection, it would be interesting to develop a SG chimera, namely a SG strain having the SE effectors, to analyze the impact on cell entry capability, on the immune response induced and thus on pathogenicity.

## Additional files

Additional file 1:
**Plasmids and primers used in this study.** Antibiotic resistance is indicated in brackets. Kanamycin (Km) was used at 50 μg mL^−1^, Chloramphenicol (Cm) at 35 μg mL^−1^, Carbenicillin (Cb) at 100 μg mL^−1^ and Tetracyclin (Tc) at 6 μg mL^−1^ [24,27,28,30,49-51]. Additional file 2:
**Results of gentamicin protection assays.** Gentamicin protection assays were performed with the 287/91, LA5 strains and their *invA* mutants after 0.5 h to 4.5 h of contact with LMH, CLEC213, DF-1, HeLa and HT-29 cells. Additional file 3:
**Complementation of** ***S*** **. Gallinarum strain 287/91 with either** ***sipA*** **,** ***sopA*** **or** ***sopE*** **from** ***S*** **. Enteritidis strain LA5 did not restore its invasive ability.** The invasive ability of *S*. Gallinarum strain 287/91 complemented with either *sipA*, *sopA* or *sopE* genes of *S*. Enteritidis was analyzed using adhesion/invasion assays performed in avian LMH cells. The experiment was carried out after 1 h of bacteria-host cell contact at a MOI of 10. Grey and black bars represent the number of adhered and intracellular bacteria and the number of intracellular bacteria respectively. The results correspond to the mean ± standard deviation of two independent experiments performed in duplicate and are expressed in log_10_ CFU mL^−1^. Similar results were obtained with avian CLEC213 cells.
