# Supplementary material for: Deciphering why Salmonella Gallinarum is less invasive in vitro than Salmonella Enteritidis
Source: Vet Res. 2014 Aug 30;45(1):81. doi: 10.1186/s13567-014-0081-z (PMC4154518; doi:10.1186/s13567-014-0081-z)
Supplement: Additional file 1: — Plasmids and primers used in this study. Antibiotic resistance is indicated in brackets. Kanamycin (Km) was used at 50 μg mL−1, Chloramphenicol (Cm) at 35 μg mL−1, Carbenicillin (Cb) at 100 μg mL−1 and Tetracyclin (Tc) at 6 μg mL−1 [24,27,28,30,49-51]. [file 13567_2014_81_MOESM1_ESM.docx]

| **Plasmids** | **Characteristics** | **Reference** |
| --- | --- | --- |
| pKD3 | Carries an FRT-Cm-FRT cassette (Cm^R^) | [24] |
| pKD4 | Carries an FRT-Km-FRT cassette (Km^R^) |  |
| pKD46 | Carries the λ-Red genes under control of P_araB_; temperature sensitive replication (Cb^R^) |  |
| pCX340 | Plasmid pCX340 (Tc^R^) carrying *β-lactamase* gene | [27] |
| pCX340sopD (pICC612) | Plasmid pCX340 carrying *sopD* *β-lactamase* fusion (Tc^R^) |  |
| pFPVmCherry | Plasmid carrying mCherry ORF (Cb^R^) (Addgene) | [28] |
| pEGFP-actin | Plasmid encoding EGFP-actin | [49] |
| pACYC177 | Plasmid pACYC177 (Km^R^ Cb^R^) | [50] |
| pACYC177*sipA* | Plasmid pACYC177 carrying *sipA* from SE LA5 (Cb^R^) | This study |
| pACYC177*sopA* | Plasmid pACYC177 carrying *sopA* from SE LA5 (Cb^R^) | This study |
| pBR322 | Plasmid pBR322 (Tc^R^ Cb^R^) | [51] |
| pBR322*sopE* | Plasmid pBR322 carrying *sopE* from SE LA5 (Tc^R^) | This study |
| **Primers** | **Sequence** | **Reference** |
| hilA-F | 5’-GGTTTAATCGTCCGGTCGTAGTG-3’ | [30] |
| hilA-R | 5’-CCTGATCCTGCATCTGAAAAGG-3’ |  |
| invF4-RT | 5’-GCGCCATCGATAAATGCCAGT-3’ |  |
| invF1 | 5’-GGTGCTGACAACTATGCTAAATACGCAGG-3’ | This study |
| sipA2 | 5’-AGGGAACGGTGTGGAGGTAT-3’ |  |
| sipA7 | 5’-CCAACGCAATGGCGAGTCAC-3’ | [30] |
| tufA-F | 5’-TGTTCCGCAAACTGCTGGACG-3’ |  |
| tufA-R | 5’-ATGGTGCCCGGCTTAGCCAGTA-3’ |  |
| invA-P1 | 5’-TTATATTGTTTTTATAACATTCACTGACTTGCTATCT  GCTATCTCACCGAGTGTAGGCTGGAGCTGCTTC-3’ | This study |
| invA-P2 | 5’-GTGCTGCTTTCTCTACTTAACAGTGCTCGTTTACGA  CCTGAATTACTGATCATATGAATATCCTCCTTAG-3’ |  |
