# Supplementary material for: Deciphering why Salmonella Gallinarum is less invasive in vitro than Salmonella Enteritidis
Source: Vet Res. 2014 Aug 30;45(1):81. doi: 10.1186/s13567-014-0081-z (PMC4154518; doi:10.1186/s13567-014-0081-z)
Supplement: Additional file 2: — Results of gentamicin protection assays. Gentamicin protection assays were performed with the 287/91, LA5 strains and their invA mutants after 0.5 h to 4.5 h of contact with LMH, CLEC213, DF-1, HeLa and HT-29 cells. [file 13567_2014_81_MOESM2_ESM.docx]

| **Cell line** | **Length of time of infection** | **Strains** | | | |
| --- | --- | --- | --- | --- | --- |
|  |  | **LA5** | **LA5*invA*** | **287/91** | **287/91*invA*** |
| **CLEC213** | **0.5 h** | 4.74 ± 0.04* | 3.92 ± 0.04 | 1.30 ± 0.23 | 0.91 ± 0.02 |
|  | **1 h** | 5.18 ± 0.07 | 4.39 ± 0.03 | 2.36 ± 0.24 | 1.57 ± 0.53 |
|  | **1.5 h** | 5.49 ± 0.03 | 4.67 ± 0.05 | 3.72 ± 0.11 | 2.74 ± 0.28 |
|  | **3 h** | 6.27 ± 0.07 | 5.64 ± 0.06 | 4.97 ± 0.15 | 4.06 ± 0.12 |
|  | **4.5 h** | 6.44 ± 0.13 | 5.74 ± 0.08 | 5.52 ± 0.10 | 4.73 ± 0.21 |
| **DF-1** | **0.5 h** | 5.10 ± 0.07 | 4.48 ± 0.09 | 1.34 ± 0.26 | 0.90 ± 0.11 |
|  | **1 h** | 5.55 ± 0.07 | 4.94 ± 0.12 | 2.32 ± 0.07 | 1.92 ± 0.46 |
|  | **1.5 h** | 6.01 ± 0.08 | 5.27 ± 0.04 | 3.48 ± 0.08 | 2.51 ± 0.26 |
|  | **3 h** | 6.67 ± 0.05 | 5.96 ± 0.07 | 4.91 ± 0.10 | 3.92 ± 0.19 |
|  | **4.5 h** | 6.90 ± 0.12 | 6.55 ± 0.13 | 5.42 ± 0.05 | 4.50 ± 0.22 |
| **LMH** | **0.5 h** | 4.86 ± 0.19 | 4.34 ± 0.25 | 1.70 ± 0.47 | 1.22 ± 0.25 |
|  | **1 h** | 5.51 ± 0.08 | 4.70 ± 0.32 | 2.15 ± 0.10 | 1.35 ± 0.25 |
|  | **1.5 h** | 5.88 ± 0.06 | 4.79 ± 0.09 | 2.52 ± 0.30 | 1.47 ± 0.44 |
|  | **3 h** | 6.45 ± 0.16 | 5.17 ± 0.05 | 4.82 ± 0.11 | 2.11 ± 0.50 |
|  | **4.5 h** | 7.08 ± 0.09 | 5.45 ± 0.01 | 5.49 ± 0.29 | 2.88 ± 0.11 |
| **HeLa** | **0.5 h** | 4.54 ± 0.21 | 3.66 ± 0.27 | 3.07 ± 0.21 | 1.42 ± 0.27 |
|  | **1 h** | 5.29 ± 0.12 | 4.02 ± 0.18 | 3.83 ± 0.36 | 1.52 ± 0.31 |
|  | **1.5 h** | 5.87 ± 0.33 | 4.28 ± 0.15 | 4.89 ± 0.16 | 1.42 ± 0.27 |
|  | **3 h** | 6.87 ± 0.09 | 4.86 ± 0.14 | 5.90 ± 0.16 | 2.43 ± 0.19 |
|  | **4.5 h** | 7.32 ± 0.22 | 5.38 ± 0.13 | 6.10 ± 0.09 | 3.04 ± 0.12 |
| **HT-29** | **0.5 h** | 3.97 ± 0.13 | 3.00 ± 0.31 | 2.44 ± 0.13 | 0.82 ± 0.39 |
|  | **1 h** | 5.07 ± 0.17 | 3.30 ± 0.20 | 2.91 ± 0.42 | 0.82 ± 0.03 |
|  | **1.5 h** | 5.61 ± 0.05 | 3.45 ± 0.30 | 3.86 ± 0.09 | 0.82 ± 0.03 |
|  | **3 h** | 6.36 ± 0.16 | 3.87 ± 0.22 | 4.78 ± 0.28 | 0.82 ± 0.03 |
|  | **4.5 h** | 6.46 ± 0.11 | 4.08 ± 0.19 | 4.98 ± 0.35 | 1.02 ± 0.25 |

* Results correspond to the mean ± standard deviation of two independent experiments performed in duplicate and are expressed in log_10_ CFU mL^-1^.
