# Supplementary material for: Deciphering why Salmonella Gallinarum is less invasive in vitro than Salmonella Enteritidis
Source: Vet Res. 2014 Aug 30;45(1):81. doi: 10.1186/s13567-014-0081-z (PMC4154518; doi:10.1186/s13567-014-0081-z)
Supplement: Supplementary file 3 [file 13567_2014_81_MOESM3_ESM.docx]

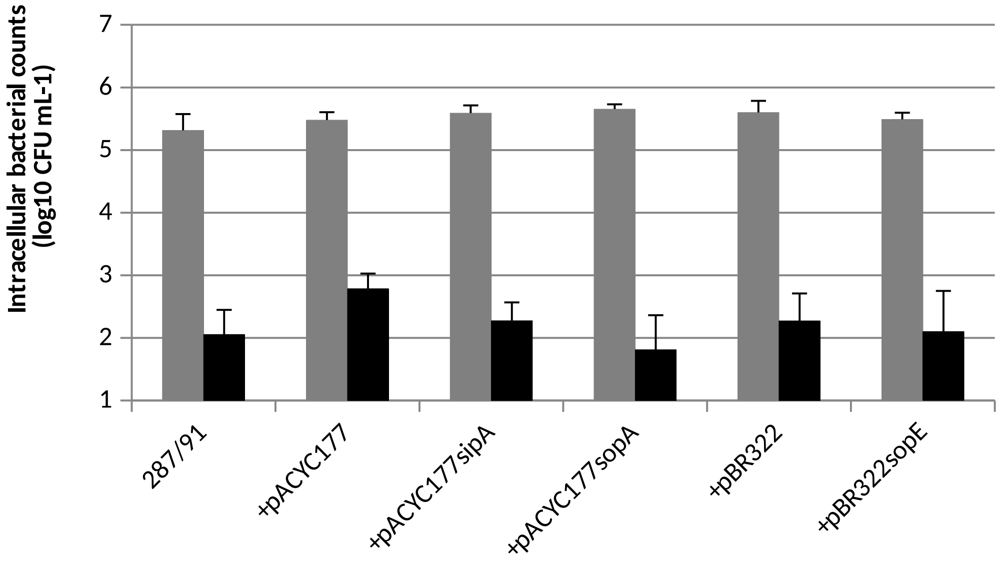

Intracellular bacterial counts (log10 CFU mL-1)
7
6
5
4
3
2
1
287/91
+pACYC177
+pACYC177sipA
+pACYC177sopA
+pBR322
+pBR322sopE
